# Postpartum Cavernous Sinus Thrombosis Associated with Eclampsia and Gestational Diabetes Mellitus: A Case Report

**DOI:** 10.31729/jnma.8904

**Published:** 2025-03-31

**Authors:** Shasank Chitrakar, Saroj Babu Aryal, Tirtha Man Shrestha

**Affiliations:** 1 Department of General Practice and Emergency Medicine, Tribhuvan University Teaching Hospital, Maharajgunj, Kathmandu, Nepal; 2 Department of Clinical Biochemistry, Tribhuvan University Teaching Hospital, Maharajgunj, Kathmandu, Nepal

**Keywords:** *cavernous sinus thrombosis*, *eclampsia*, *gestational diabetes mellitus*, *rivaroxaban*

## Abstract

Pregnancy is the pro-thrombotic state. The objective of this report is to highlight the rare occurance of postpartum cavernous sinus thrombosis associated with eclampsia and gestational diabetes mellitus, emphasizing its clinical presentation. A 25-year-old Muslim female came with the complaint of headache,post auricular and right parietal region on 8th day post-partum. She had a history of postpartum eclampsia and Gestational Diabetes Mellitus for which she was treated with magnesium sulfate and oral hypoglycaemic agents respectively. MRV showed a filling defect in right transverse sinus, sigmoid sinus, and internal jugular vein. She was then admitted to the ward and was treated with heparin and antibiotics. She was then discharged on oral rivaroxaban. As pregnancy is itself a state of hyper-coagulable state, there seems to be a significant thrombosis risk. Hence clinicians should be aware of the possible thrombotic disorder in the background of this vignette.

## INTRODUCTION

Cavernous sinus thrombosis is a life-threatening disorder that complicates facial infection, sinusitis, orbital cellulitis,or otitis following trauma or surgery, especially in thrombophilic state ^1^. It has high mortality and morbidity rates ^2^. Staphylococcus aureus are the most common cause of septic thrombosis of the cavernous sinuses ^3^. The risk of primary thrombotic events was markedly higher within 6 weeks ^4^. The incidence of VTE has been reported to be highest immediately after childbirth ^5^. In pregnant women or up to 6 months in the postpartum, global incidence was found to be around 0.1% ^6^. This case highlights the rare clinical entity, diagnostic challenges and therapeutic implications.

## CASE REPORT

A 25-year-old married female, non-alcoholic or nonsmoker, non-vegetarian presented to the emergency department referred from Janakpur Hospital with chief complaints of right-sided post auricular pain and headache for the last 5 days. Headache was located at the right parietal region, sudden onset, throbbing, continuous type, non-radiating and there was no associated photophobia, eye pain, lacrimation, visual blurring, loss of consciousness, or limb weakness. There was no history of nausea, vomiting, or trauma to the head. She was a regularly menstruating woman with normal cycles. It was her 8^th^ post-partum day when she developed the symptoms mentioned above.

She was previously diagnosed with Gestational Diabetes Mellitus during her antepartum period and was started on Metformin twice daily. Her pregnancy was uncomplicated till one hour postpartum, following normal vaginal delivery, when she developed Generalised Tonic Clonic Seizures with features like stiffening of bilateral upper and lower limbs, frothing from the mouth, neck deviated to the left side, shaking vigorously, and up rolling of eyes. Her seizure lasted for 20 seconds followed by a period of post-ictal confusion state and drowsiness. She was then diagnosed with a case of Post-partum Eclampsia and treatment of presumed postpartum eclamptic seizure was started at 6g(8mL) bolus of intravenous magnesium sulfate diluted 12mL of normal saline slowly over 20 minutes. She was admitted to the ward for a few days and was discharged.

**Figure 1:**
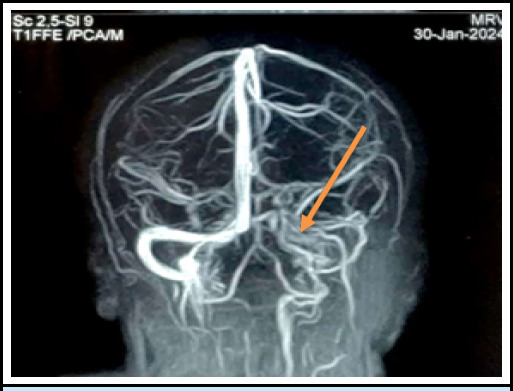
MRV showing filling defect in right transverse sinus, sigmoid sinus, and inferior vena cava

At her hospital admission this time, she looked ill. Her pulse rate was 106 beats/min, regularly regular with a blood pressure of 130/80 mm Hg. Her respiratory rate was 20 breaths/min with SpO2 of 94% in room air and temperature of 97.2°F. All other systemic examinations were normal. Her neurological examination was also normal with no limb weakness. There was no neurological deficit noted at the time of examination.

**Figure 2:**
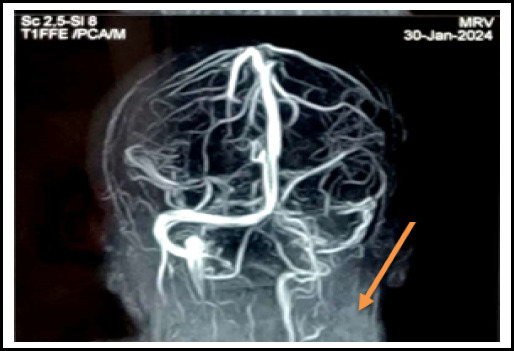
MRV showing filling defect in right transverse sinus, sigmoid sinus, and inferior vena cava

Initial routine blood investigations including Thyroid function test was normal. Electrocardiography showed normal sinus rhythm. But her Magnetic Resonance Imaging Brain with Magnetic Resonance Venography gave the impression of a "Filling defect in Right transverse sinus and sigmoid sinus as well as internal jugular vein likely thrombosis" (Figure 2).

Based on clinical history, physical examinations, laboratory investigations, and imaging, the diagnosis of Right-Sided Cavernous Sinus Thrombosis was made and she was planned for medical treatment with low molecular heparin, oral antibiotics, and oral anticoagulants. Her biochemical parameters one day before discharge were accessed and were found to be within normal range. The patient was admitted for 5 days and then was discharged on oral medications.

She was then assessed after 15 days following discharge. She was found to be in good health. Her headache had subsided significantly and there was no bleeding complication due to her ongoing drug therapy. She has been advised to do routine follow-ups.

**Figure 3:**
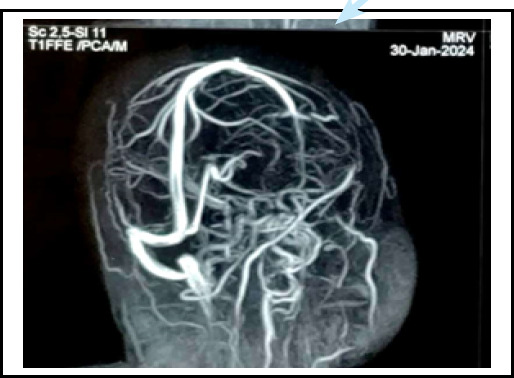
MRV showing filling defect in right transverse sinus, sigmoid sinus, and inferior vena cava

## DISCUSSION

Coagulation is the process by which thrombin is activated and soluble plasma fibrinogen is converted into insoluble fibrin.^7^ These steps account for both normal hemostasis and the path of physiologic processes influencing the development of venous thrombosis.^7^ The risk factors for venous thrombosis are primarily related to hypercoagulability, which can be genetic or acquired, or due to immobilization and venous stasis.^7^

Cerebral Venous Thrombosis is an uncommon illness that has a critical outcome.^8^ The rate from 0.018% to 0.2% had been reported for developing venous thrombosis during pregnancy and puerperium.^8^ The prevalence of Cerebral Venous Thrombosis has long been likely to be about 0.3-0.5/100,000 per year; nevertheless, the latest studies have declared a greater level of around 1-1.5/100,000 per year.^8^

Cavernous sinus contains oculomotor nerve, trochlear nerve, an ophthalmic branch of the trigeminal nerve, maxillary branch of trigeminal nerve, internal carotid artery, abducens nerve, trochlear nerve.^9^

MR cerebral venography is a Magnetic Resonance Imaging examination of the head with either contrast-enhanced or non-contrast sequences to assess the patency of the Dural venous sinuses and cerebral veins.^10^ Suspected cerebral venous thrombosis is the primary indication.^10^ Magnetic Resonance Imaging can visualize the clot as well as the sequelae.^11^ The clot acutely is isointense on T1 and hypo intense on T2 (this can mimic a flow void), with the sub-acute clot becoming hyper-intense on T1.^11^

We report the case of a 35-year-old female, para 8 and gravida 8, brought to the emergency department on her ninth day postpartum, having severe headaches and confusion; during her emergency admission, she suffered two episodes of generalized seizure. On admission, the patient's blood pressure was 200/120 mmHg. The patient was first diagnosed with postpartum eclampsia and managed with magnesium sulfate for seizures and hydralazine for blood pressure control. Onward admission, she continued having seizures and her level of consciousness decreased with left-side weakness. An urgent MRI of the head and Magnetic Resonance venography performed immediately revealed acute thrombosis involving the anterior aspect of the superior sagittal sinus and the left transverse sinus. The patient was moved to the Intensive Care Unit. Anti-coagulant Low Molecular Weight enoxaparin and anticonvulsant were started.^8^

A retrospective analysis conducted in a neurology clinic in Romania from 2009 to 2020 concluded that "The early postpartum period represents an important risk for the development of CVT (Cerebral Venous Thrombosis). Cesarean delivery and preeclampsia, besides general risk factors such as infection, smoking, and primary thrombophilia, contribute to enhanced risk. Puerperium-related CVT presents a more favorable outcome compared with CVT with other aetiologies".^12^

A prospective study conducted at Koppal Institute of Medical Sciences, Koppal, Karnataka, India 2012 to 2015 concluded that "Pregnancy and puerperium are most prevalent prothrombotic states leading to CVT".^13^

## CONCLUSIONS

Cerebral venous thrombosis is a life-threatening hypercoagulable state. The clinical presentation of cerebral venous thrombosis often resembles a simple headache and is often misdiagnosed. Imaging plays a primary role in diagnosis with magnetic resonance imaging (MRI) and magnetic resonance Venography being the diagnostic modality of choice.
